# The Impact of Integration of E-Learning in Science: Applying Technology in Biology Classes as a Model

**DOI:** 10.7759/cureus.60646

**Published:** 2024-05-20

**Authors:** Nada K Abuarab, Ahmed A Aldarmahi

**Affiliations:** 1 College of Science and Health Professions, King Saud Bin Abdulaziz University for Health Sciences, Jeddah, SAU

**Keywords:** teaching methods, pre-professional year, lecturing, e-learning, education

## Abstract

Background

The most common teaching method in science is the lecture-based approach. E-learning is one of the advanced teaching methodologies that have gained attention in recent decades. It was hypothesized that the intervention of e-learning improved students’ scores and satisfaction in biology classes. The purpose of this study was to determine if e-learning combined with traditional learning methods of lectures would improve unified students' scores and satisfaction in biology classes.

Methodology

The study design was a quasi-experimental approach. This study was conducted at the College of Science and Health Professions Jeddah, King Saud bin Abdulaziz University for Health Sciences, Jeddah, Kingdom of Saudi Arabia. The study subjects comprised first-year female students (pre-professional program) in two different classes. The total number of participants was 150 (75 students in each group). The systematic random sampling technique was used. Randomly, one class (experimental group) received e-learning in addition to traditional lectures, and the other group (control group) received traditional lecture. The “Biology for Health Sciences” course was selected for this purpose. Scientific pre-test and post-tests were performed, and a satisfaction questionnaire was filled in for both groups.

Results

We found that e-learning and traditional lecture improved students’ scores (P<0.01). Learning progress in the e-learning group was significantly more than that in the control group (P<0.05). Lecture has been shown to increase learning gains by 33%, and the integration of e-learning increased learning gains by 62%. Analysis of questionnaire results showed improved student satisfaction with the course in the study group.

Conclusion

The integration of e-learning approach significantly improved the retention and students’ scores and satisfactions. E-learning could be applied more in pre-professional year science courses. According to these advantages, the quality of science education can be improved with this approach.

## Introduction

The use of traditional teaching (lecturing) is an effective way to communicate a large amount of information to learners [1]. It is cost-effective as teachers can reach out to many listeners at one time [1]. However, some educational objectives cannot be achieved by lecturing. This leads to a gap between teaching and learning [2]. A study by Moravec et al. showed that the potential key to eliminating this gap is using modified lectures as they play an important role in achieving a wide range of learning outcomes [3].

The modified teaching method has provided a model for incorporating alternative (nonlecture-based) teaching methodologies such as learning on computers, case studies, writing in class, and problem-solving [3]. The use of computers and technologies to deliver knowledge plays an important role in teaching and learning. It is a recent trend and is referred to as the “e-learning revolution” [4]. E-learning is an abbreviation of the term “electronic learning.” It is facilitated by electronic media such as audio or visual tapes [4,5].

E-learning also refers to a combination of training methods, namely classroom teaching, multimedia modules (text, graphics, animation, audio, and video), and online chatrooms [4,5]. It is the most promising educational technology as it is cost-effective, is fast, is of high quality, is easily updatable, and encourages retention [4,6].

It provides an active environment for learners by using interactive videos. The videos provide teachers with the opportunity to interact with learners and provide feedback. However, there are disadvantages of using e-learning, as it may not be suitable for all disciplines and requires technical support, trained teachers, and experts [6,7].

Several studies have indicated that e-learning has made significant contributions to medical education [6]. Other studies showed that integration of e-learning led to improvement in students’ acquisition of basic knowledge in evidence-based medicine internship training courses compared to traditional lecture-based courses [8]. However, there are a limited number of studies that have evaluated the effectiveness of e-learning introduced alongside traditional methods.

The College of Science and Health Professions (COSHP), King Saud bin Abdulaziz University for Health Sciences (KSAU-HS), Jeddah, Kingdom of Saudi Arabia, provides a one-year preparatory program to assist students in preparing for a full multi-year medical degree curriculum and offers a bridge between the students’ high-school studies and university-level studies. The preparatory year also aims to help the students transition from the high school system of teaching/learning to that of the university and to acquaint the students with the various academic disciplines. The Department of Basic Science of COSHP provides a number of science courses, such as Biology for Health Sciences, Chemistry for Health Sciences, Physics for Health Sciences, and Biochemistry. Biology for Health Sciences (BIOL 101) is an introductory module that aims to present the main concepts of biology to students, including cell structure and function, cell cycle, and basic molecular biology.

In COSHP, the instructional method used in classes is the traditional lecture. Traditional teaching is an effective way to achieve instructional goals. However, this information is easily forgotten by disengaged students. Integrating e-learning into a course curriculum will be valuable in increasing students’ understanding of difficult concepts, improving students’ performance, and decreasing the failure rate. As a result, the purpose of the study is to evaluate the effectiveness of the integration of e-learning methods for teaching biology to first-year students compared to a traditional lecture-based biology course with equivalent content.

The theoretical framework and assumptions of the study are based on Kirkpatrick’s four levels of training evaluation [9]. This theory serves as the basis for conducting this research study, and it is a highly useful model in evaluating both students’ satisfaction and learning outcomes (level 1 and level 2) with and without the intervention.

The key aim of the study is to test the hypothesis outlined above by determining if e-learning combined with traditional learning methods of lectures would improve female students' scores and satisfaction for first-year students over those who experienced traditional learning alone in biology classes using adjunct e-learning at COSHP. Specific objectives were taken to achieve the aim of the study, which are to evaluate the effectiveness of the integration of e-learning methods on student achievement by comparing the control group with the experimental group and to measure students’ level of satisfaction with the education methods used in the biology classroom.

## Materials and methods

The experimental design for this study is a quantitative experimental research study (intervention, comparing the control group with the experimental group), which is based on the following hypotheses: null hypothesis, in which the intervention of e-learning is not associated with students’ scores and satisfaction, and alternative hypothesis, in which the intervention of e-learning is associated with students’ scores and satisfaction.

Students were arbitrarily divided into two groups. In one group, the students were exposed to PowerPoint presentations based on traditional lectures. While in the second group, the students were exposed to e-learning methods (using videos in the classroom) introduced alongside lectures with equivalent content to the first group. The traditional lectures were course content delivered via the instructor’s spoken presentation where the students listened without active engagement. On the other hand, in the e-learning method, the instructor relied on lectures, presentations, and videos to deliver the course content to the students. After the students watched the videos, they discussed questions related to the lecture and interacted with course content. Both the groups had a pre-test in the classroom followed by a post-test after the lecture within a semester period (15 weeks). Student performance and change were compared within the two groups. The selected lecture topics for this study were cell cycle, DNA replication and repair, and DNA transcription. A satisfaction questionnaire was used to measure overall students’ satisfaction.

Study area/setting

This study was conducted in the Biology for Health Sciences (BIOL 101) classroom setting at the Department of Basic Science on the female-only campus of COSHP. BIOL 101 is a course with one hour of lecture per week and two hours of lab per fortnight over a 15-week semester.

Study subjects

The sample was composed of KSAU-HS unified first-year female students (undergraduate students) aged 18-20 years who were studying in the Department of Basic Science on the female-only campus and taking BIOL 101.

Sample size

The total number of undergraduates was 235 students in COSHP. The total accessible number of students was 150 (whole batch of unified students).

The required sample size was calculated via Open Epi software by considering the following assumptions: significance level as 0.05 and confidence level 95%. Therefore, the total calculated sample size was 55 (each group). The values illustrated above were estimated based on the previous study [10]. However, keeping in view the non-response rate and dropout rate, we included all the accessible students of first-year COSHP, i.e., 150 students (75 in each group).

Sampling technique

Systematic random sampling technique was used to select the subjects of the control group and experimental group. As it is important that the group selected be representative of the population, ordering sampling list alternates between the control group (on the even numbers) and experimental group (on the odd numbers).

Study design

The study used a quasi-experimental non-equivalent control group design (data collected before and after the intervention). The justification of this study design is that this research study aimed to compare student’s performance between the experimental group and control group and to assess the effect of the intervention (e-learning) on the sample with confidence [11]. This study design is in alignment with level 2 Krikpatrick’s model (learning: pre- and post-test) [9]. Therefore, Krikpatrick’s four-level framework was used as the guiding framework for this study and as a starting point [9].

Study tools and process

The pre- and post-test designs were the preferred method to compare participant groups and measure the degree of change occurring because of interventions. The questions in the pre-test will be the same questions in the post-test. As validity is an important feature of an instrument [12], the test was validated using content validity; all the items were reviewed by experts in biology for validation to make sure the test measures what it is supposed to measure and to compare the content of the test to the domain [13]. The test contained 15 objective items. Each objective item was allocated 10 points. The time allocated for the test was 15 minutes. The selected lecture topics for this study were cell cycle, DNA replication and repair, and DNA transcription.

The students were asked to indicate to what extent they agreed or disagreed with a series of statements. A validated questionnaire was used to measure overall students’ level of satisfaction with the education methods used [14].

Data management and analysis plan

The data were entered and analyzed using SPSS Version 24.0 (IBM Corp., Armonk, NY). Descriptive and inferential tests were used to compare outcomes. Statistical analysis was performed using the independent t-test, paired t-test, and chi-square test. A p-value of less than 0.05, 0.01, or 0.001 was considered as statistically significant difference for all of the statistical tests.

## Results

An examination of the effect of traditional lecture and e-learning methods on students’ achievement

**Figure 1:**
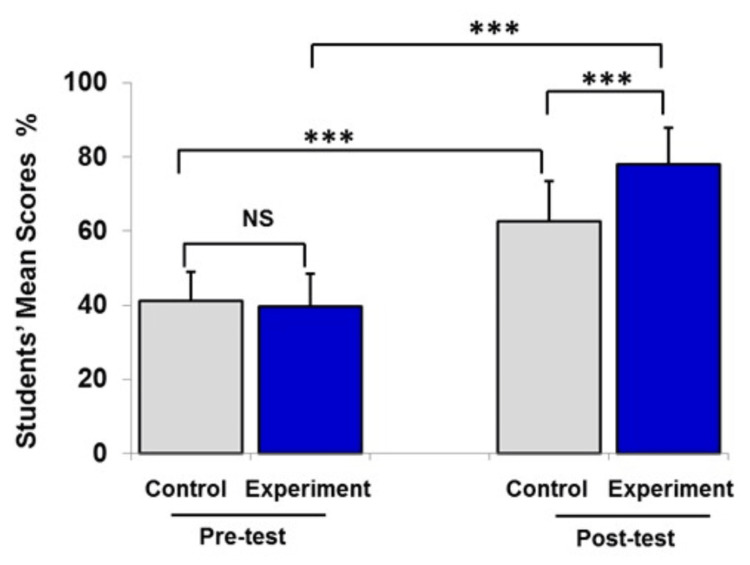
Pre- and post-test mean scores of the experimental group and the control group after the cell cycle lecture. Data are presented as mean ± SEM of percentage of students’ mean scores (%). Number of students in the experimental group was 75 and that in the control group was 75. Statistical analysis was performed using the independent t-test and paired t-test. ***P<0.001 NS, no significant difference between groups

**Figure 2:**
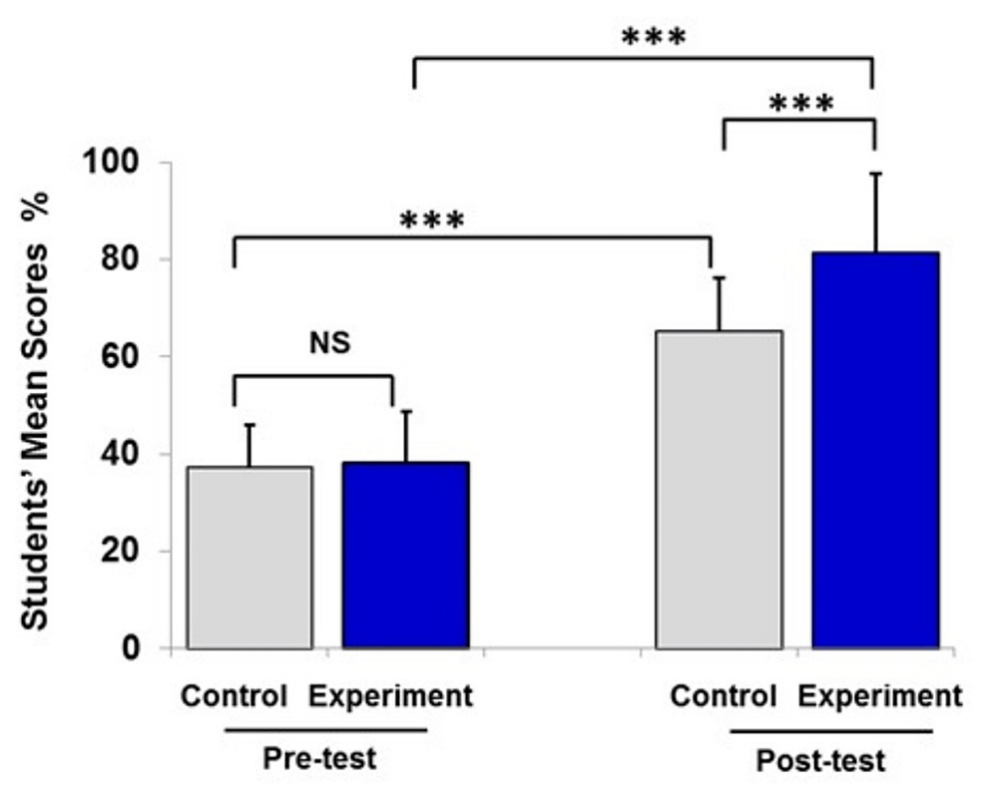
Pre- and post-test mean scores of the experimental group and the control group after the DNA transcription lecture. Data are presented as mean ± SEM of percentage of students’ mean scores (%). Number of students in the experimental group was 75 and that in the control group was 75. Statistical analysis was performed using the independent t-test and paired t-test. ***P<0.001 NS, no significant difference between groups

**Figure 3:**
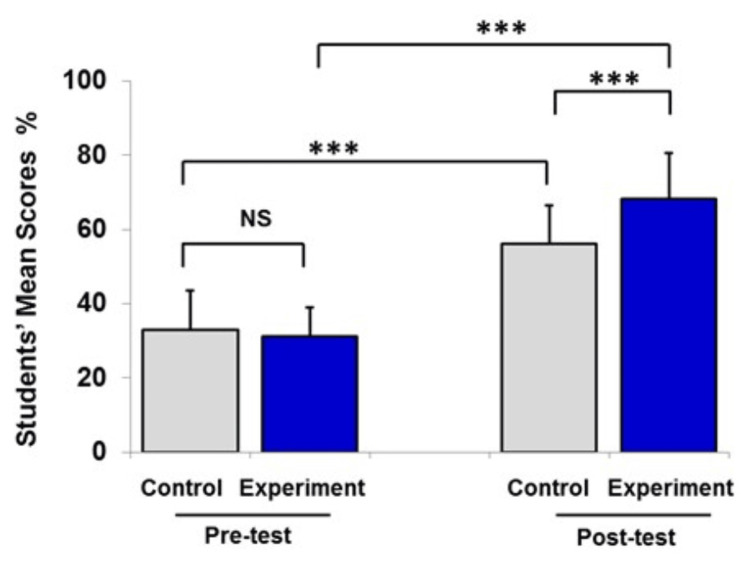
Pre- and post-test mean scores of the experimental group and the control group after the DNA replication and repair lecture. Data are presented as mean ± SEM of percentage of students’ mean scores (%). Number of students in the experimental group was 75 and that in the control group was 75. Statistical analysis was performed using the independent t-test and paired t-test. ***P<0.001 NS, no significant difference between groups

As seen in the statistical results in Figure 1, it was found that the pre-test results were not statistically significantly different between the control group and the experimental group, with a mean of 61.7±11.6 and 59.6±12.9, respectively. However, the bar chart displayed that there was a significant increase in students’ post-test mean scores (~21%) in the control group after the cell cycle lecture (P<0.001). This means there was an increase in students’ learning level after the lecture. Moreover, the post-test mean scores of the experimental group increased by 38.4%. The experimental group scored significantly higher than the control group (P<0.001). Experiment was independently repeated three times and yielded similar results (Figures 2, 3).

The statistical analysis in Figure 2 showed that the mean students’ score in the pre-test for the study group and the control group was 37.2% and 38.2% respectively. There was a significant difference between the scores of the study group (81.5%±16) and the control group (65.1%±11) in the post-test (P<0.001).

Analysis of students’ scores in the DNA replication and repair lecture revealed insignificant differences between the two groups in the pre-test (before the intervention) (Figure 3). The students were assessed after the intervention, and data indicated that the difference between the achievement’s mean scores for the experimental and control groups was significant (P<0.001).

**Figure 4:**
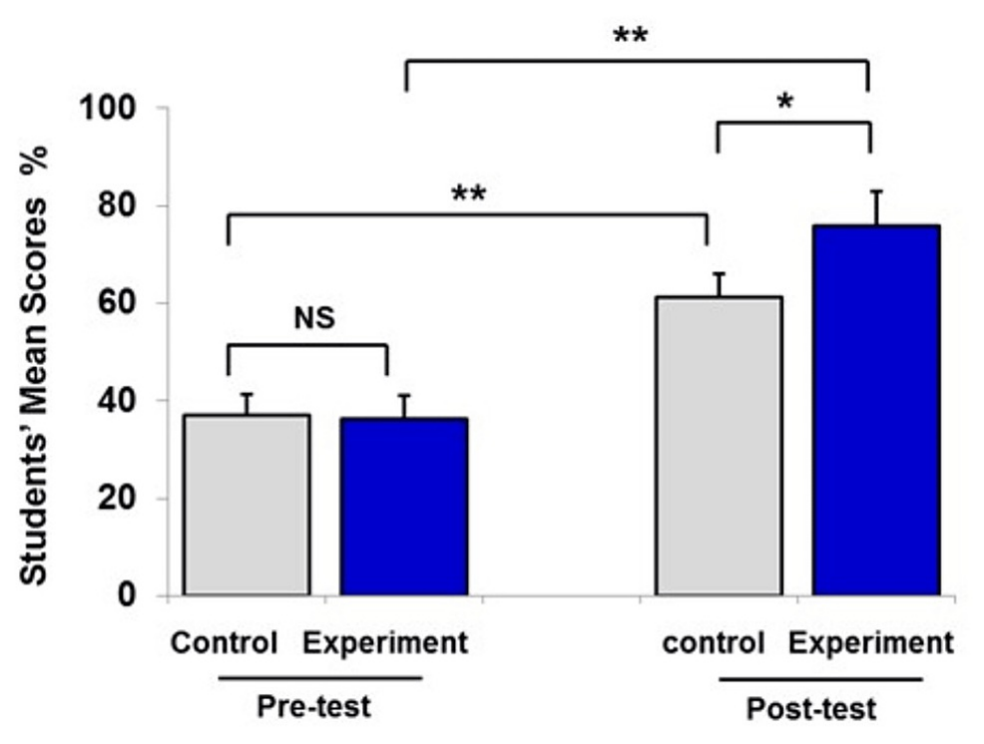
Pre- and post-test mean scores of both groups. Data from Figures 1-3 are combined and presented as mean ± SEM of percentage of students’ mean scores (%). Number of students in the experimental group was 75 and that in the control group was 75. Number of experiments was 3. Statistical analysis was performed using the independent t-test and paired t-test. *P<0.05, **P<0.01 NS, no significant difference between groups

Data from Figures 1-3 were expressed as mean ± SEM, and in Figure 4 (combined data), the control and experimental groups were at the same level of achievement at the start of the study (37±4%, and 36±4%, respectively). The traditional teaching method (lecturing) indicated a significant improvement of students’ learning level (P<0.05). However, integration of instructional videos with lecture in the classroom had a significant positive impact on students’ scoring compared to lecturing only.

Calculating learning gain scores

**Figure 5:**
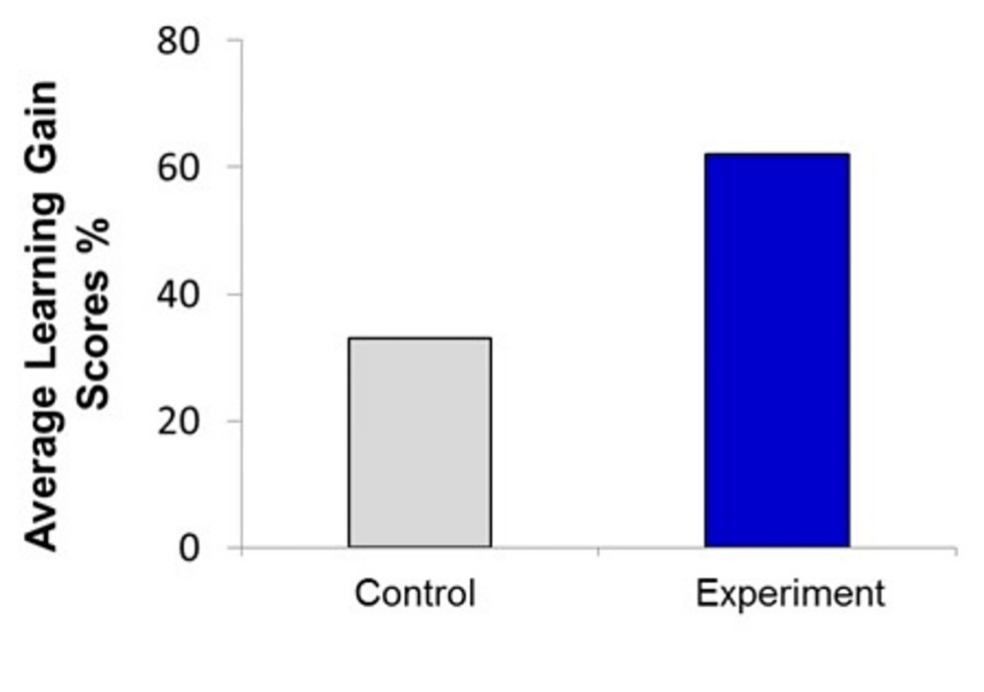
Average of learning gain. Statistical analysis is presented as average learning gain (%) in the control group and the experimental group. Number of students in the experimental group was 75 and that in the control group was 75. Statistical analysis was performed using the independent t-test P=0.012 (statistically significant).

The average learning gain scores were calculated for each group using the formula: (post-assessment - Pre-assessment) / (100% - Pre-assessment) x 100 [15]. Average of learning gain from lecture was 33%, and that from lecture in the presence of e-learning was ~62%. The using of e-learning in biology classes improved students’ learning gain by ̴~29%, as depicted in Figure 5.

The effect of teaching method on students' satisfaction in the control and study groups

The students’ satisfaction questionnaire response rates were 100% in the control group and 86% in the study group.

Statements 2, 3, 6, 7, and 8 regarding the performance feedback, their engagement in learning, the fairness and transparency of the assessment, their level of satisfaction with the quality of the components including lectures and videos (experimental group only), and the effectiveness of course in their learning indicated that no relationship exists between the two variables (teaching method categories and the statement). In response to statements 1 and 4, the strongest relationship was found between the organization of the course and effectiveness of lecture delivery, and teaching methods. This relationship was statistically significant at P<0.05 and P<0.01. Statement 5 was regarding the effectiveness of availability of online teaching materials in their learning. It was found that 90% of the students agreed to online resources being useful in their learning (Table 1). Consequently, the results indicated that both groups reported predominantly positive responses with a high level of satisfaction. Additionally, a statistically significant correlation was found between teaching methods and satisfaction regarding course organization (control group=56%, experimental group=72%), as well as the effectiveness of the delivery method used in the class (control group=77%, experimental group=81%).

**Table 1:** Students’ perceptions of teaching methods (component) used in the biology class in the control and study groups. Data are presented as mean of agreement (%). There were 75 students in the control group (100% response rate) and 65 students in the study group (86% response rate). Neutral survey responses were excluded from statistical analysis as they did not represent genuine preferences. Statistical analysis was performed by independent t-test and paired t-test. *P<0.05, ***P<0.001. †This question was asked only in the control group (no online video interventions). NS, no significant difference between groups

Question	Number		Agreement %		P-value
	Control	Study	Control	Study	
1. This component was well-organized.	25	35	56.81%	72.91%	0.000292***
2. I received helpful feedback and support.	29	29	69.04%	76.31%	0.182 (NS)
3. This component engaged me in learning.	32	34	72.72%	79.06%	0.29 (NS)
4. The teaching on this component was effective in helping me learn.	34	36	77.77%	81.81%	0.024*
5. Learning material would be more effective in helping me learn biology if available online.^†^	45	-	90%	-	-
6. The biology assessment test was clear and fair.	29	32	69.04%	71.11%	0.595 (NS)
7. Overall, I am satisfied with the quality of this component.	26	33	66.66%	76.74%	0.122 (NS)
8. Overall, how effective was this component in helping you to learn? (effective, N (%))	44	42	59.09%	64.51%	0.093 (NS)

## Discussion

This study aimed to determine if e-learning combined with traditional learning methods of lectures would improve female students' scores and satisfaction for first-year students over those who experienced traditional learning alone in biology classes. Therefore, Kirkpatrick's program evaluation model is more appropriate for this study, and it was used to evaluate students' scores and satisfaction [9,10].

The findings revealed the value of traditional classrooms for learning effectiveness, as was clearly obtained in the results. Students in the e-learning that provided videos achieved significantly better learning performance action than those in traditional classrooms. The highest score of the control group was 80%, and that of the experimental group was 100%. As a result, the lecture method is an important method to improve students’ learning. However, it may be essential to integrate instructional videos into traditional classrooms for better learning performance.

Pervious study found that modified lectures increased student attendance and engagement, and the gain learning was more than twice (physics classroom) [16]. Similarly, another study found that using self-regulated e-learning modules represents a novel instructional approach aimed at enhancing student achievement and results [17]. Moreover, a study conducted by Issa et al. showed that using multimedia (e-learning) has a significant contribution to medical education and enhanced students’ short-term retention [18]. Another study found that the development of e-learning contributes to enhancing not only knowledge but also students' skills [19]. As a result, these research study’s findings are in agreement with other previous studies showing the positive effects of using modified lectures and e-learning on students’ performance.

In science courses, as the lecture-based format may not be the most efficient way for all teaching objectives, modifying lectures by including multimedia, case studies, and problem-solving techniques would improve students’ learning compared to traditional lectures [4].

As seen from learning gain data in Figure 5, we examined the relationship between the teaching methods used in the class and student learning gain in the three different subject areas. The results suggest that e-learning is associated with greater learning gain. Assessment of the second level of Kirkpatrick’s model revealed a significant contrast in the participants’ learning scores prior to and following the intervention. However, the results may be composed of other factors such as e-learning boosts students' interest, leading to improved students' learning.

A recent study found that students were almost satisfied and in favor of using technology when they were entering teaching and learning [20]. The outcome of the reaction part (level 1 of Kirkpatrick's program) indicated that the participants expressed satisfaction. These days, using information technology to enhance learning is widely appreciated [14,20,21]. Findings of this study were consistent with previous study findings that indicated the use of e-learning resulted in students’ satisfaction and engagement [14].

Limitations of the study

The first limitation is the limited number of enrolled female students in the college. The second limitation is that this study examined the effectiveness of e-learning in the biology course only, and the results might be inapplicable to other courses. However, other science courses can provide a comprehensive view of the effectiveness of e-learning in science.

## Conclusions

The purpose of this study was to determine if e-learning combined with traditional learning methods of lectures would improve unified female students' scores and satisfaction in biology classes at KSAU-HS. The presence of E-learning in the traditional biology classroom is achievable and resulted in more effective learning gain in comparison to the lecture-only method. Students in the e-learning group achieved significantly better learning performance. Analysis of survey results showed improved student satisfaction with the course in the experiment group. These findings suggest that the use of e-learning tools such as instructional videos for teaching biology could be an effective supplement for improving students’ scores and satisfaction. However, further studies and additional educational tools are needed to generalize the findings of this study with consideration of other factors that should be recognized to implement a successful technique to modify the lecture. E-learning could be applied more in undergraduate science courses. According to these advantages, the quality of science education can be improved via this method.

## References

[1] Chism NV, Cano J, Pruitt AS (1989). Teaching in a diverse environment: knowledge and skills needed by TAs. New Dir Teach Learn.

[2] Zhao B, Potter DD (2016). Comparison of lecture-based learning vs discussion-based learning in undergraduate medical students. J Surg Educ.

[3] Moravec M, Williams A, Aguilar-Roca N, O'Dowd DK (2010). Learn before lecture: a strategy that improves learning outcomes in a large introductory biology class. CBE Life Sci Educ.

[4] Salehi H, Shojaee M, Sattar S (2014). Using e-learning and ICT courses in educational environment: a review. Canadian Center Sci Educ.

[5] Zhang D, Nunamaker JF (2003). Powering E-learning in the new millennium: an overview of e-learning and enabling technology. Inf Syst Front.

[6] Packham G, Jones P, Miller C, Thomas B (2004). E‐learning and retention: key factors influencing student withdrawal. Educ Train.

[7] Nawaz A, Khan MZ (2012). Issues of technical support for e-learning systems in higher education institutions. Int J Mod Educ Comput Sci.

[8] Hadley J, Kulier R, Zamora J (2010). Effectiveness of an e-learning course in evidence-based medicine for foundation (internship) training. J R Soc Med.

[9] Kirkpatrick DL (2006). Seven keys to unlock the four levels of evaluation. Perform Improv.

[10] Uzun A, Şentürk A (2010). Blending makes the difference: comparison of blended and traditional instruction on students' performance and attitudes in computer literacy. Contemp Educ.

[11] Grimshaw J, Campbell M, Eccles M, Steen N (2000). Experimental and quasi-experimental designs for evaluating guideline implementation strategies. Fam Pract.

[12] Considine J, Botti M, Thomas S (2005). Design, format, validity and reliability of multiple choice questions for use in nursing research and education. Collegian.

[13] Almanasreh E, Moles R, Chen TF (2019). Evaluation of methods used for estimating content validity. Res Social Adm Pharm.

[14] Ariana A, Amin M, Pakneshan S (2016). Integration of traditional and e‐learning methods to improve learning outcomes for dental students in histopathology. J Dent Educ.

[15] Andrews TM, Leonard MJ, Colgrove CA, Kalinowski ST (2011). Active learning not associated with student learning in a random sample of college biology courses. CBE Life Sci Educ.

[16] Deslauriers L, Schelew E, Wieman C (2011). Improved learning in a large-enrollment physics class. Science.

[17] Logan RM, Johnson CE, Worsham JW (2021). Development of an e-learning module to facilitate student learning and outcomes. Teach Learn Nurs.

[18] Issa N, Schuller M, Santacaterina S, Shapiro M, Wang E, Mayer RE, DaRosa DA (2011). Applying multimedia design principles enhances learning in medical education. Med Educ.

[19] Supriyatno T, Susilawati S, Hassan A (2020). E-learning development in improving students' critical thinking ability. Cypriot J Educ Sci.

[20] Al-Janabi S, Huisman A, Van Diest PJ (2012). Digital pathology: current status and future perspectives. Histopathology.

[21] Hamilton PW, Wang Y, McCullough SJ (2012). Virtual microscopy and digital pathology in training and education. APMIS.

